# Professional identity formation of medical students: A mixed-methods study in a hierarchical and collectivist culture

**DOI:** 10.1186/s12909-022-03393-9

**Published:** 2022-06-08

**Authors:** Ardi Findyartini, Nadia Greviana, Estivana Felaza, Muhammad Faruqi, Taris Zahratul Afifah, Mutiara Auliya Firdausy

**Affiliations:** 1 Medical Education Center, Faculty of Medicine, Indonesia Medical Education and Research Institute, Universitas Indonesia, Jakarta, Indonesia; 2 Department of Medical Education, Faculty of Medicine Universitas Indonesia, Jakarta, Indonesia; 3 Undergraduate Medical Program, Faculty of Medicine, Universitas Indonesia, Jakarta, Indonesia

**Keywords:** Professional identity formation, Medical students, Mixed-methods study

## Abstract

**Background:**

Professional identity formation (PIF) has been recognized as an integral part of professional development in medical education. PIF is dynamic: it occurs longitudinally and requires immersion in the socialization process. Consequently, in the medical education context, it is vital to foster a nurturing learning environment that facilitates PIF.

**Aim:**

This study assesses PIF among medical students in various stages of study and explores their perceptions of PIF, with its contributing and inhibiting factors.

**Method:**

This mixed-methods study uses a sequential explanatory approach with undergraduate (years 2, 4, and 6) and postgraduate medical students in Indonesia. We examine the subjects by administering an adapted questionnaire on PIF. We completed a series of FGDs following questionnaire administration. Quantitative and thematic analyses were conducted sequentially.

**Results & Discussion:**

A total of 433 respondents completed the questionnaire. There were statistically significant differences among subjects on the subscales “Recognition and internalization of professional roles” and “Self-control in professional behavior”; the more senior students had higher scores. We conducted 6 FGDs in total. The results characterize PIF as a complex, dynamic, and longitudinal journey to becoming a medical doctor that is closely related to a student’s motivation. The FGDs also highlight the importance of both internal factors (students’ values, attributes, and personal circumstances) and external factors (curriculum, the learning environment, workplace-based learning, and external expectations) for PIF in medical education.

**Conclusion:**

Higher-level students show higher scores in some aspects of PIF, which further validates the potential use of the questionnaire to monitor PIF, a dynamic process influenced by internal and external factors. Generating awareness among medical students and encouraging reflection on their PIF stage may be crucial for PIF processes.

**Supplementary Information:**

The online version contains supplementary material available at 10.1186/s12909-022-03393-9.

## Introduction

Medical professionalism encompasses multiple behaviors that may change over time [1] and requires the professionals to picture themselves as the member of the medical professions who are able to provide excellent, ethical and altruistic patient care [2]. It stands on basic principles such as excellence, accountability, altruism, and humanism [3], as well as adherence to ethical principles, effective interactions with patients and their family members, effective interactions with the healthcare system, and commitment towards improvement for self, others and the system [4]. It has been further emphasized that virtue-based and behavior-based professionalism in medicine should be strengthen by personal and professional identity formation [5].

One way to anchor professional development is to have students recognize their own professional identity formation [2, 5]. This formation begins when they become medical students and continues after their graduation. A wide range of interactions with teachers, peers, senior colleagues, and the broader medicine/healthcare community immerses medical students in the socialization process [6], which is central to PIF [7]. Consequently, a supportive community of practice and a nurturing learning environment are important for medical students to internalize professional attitudes [8]. In a clinical setting, professional identity helps healthcare professionals define practice boundaries and reduce role confusion in teamwork [9]. Thus, it facilitates the advocacy of professional opinions for both practitioners [10] and educators [11]. A scoping review on PIF among medical students highlights that it is a multifactorial phenomenon which involves a continuous construction and deconstruction of individual, relational and societal identities. This dynamic nature can be influenced by individual values and beliefs and their interactions with environmental factors including clinical and non-clinical experiences of medical students [12].

In medical education, Kegan’s model is acknowledged by researchers as deeply analyzing the PIF process in medical students. Based on this model, we categorize PIF into 6 stages: the incorporation, impulsion, imperial, interpersonal, institutional, and inter-individual stages. It is believed that students undergo stages 2–4 during medical education [7]. In stage 2 (the imperial stage), medical students are expected to recognize and follow professional rules without adequate self-reflection. As PIF advances in stage 4 (the institutional stage), medical students develop to understand relationships in terms of different values and expectations. Eventually, they become more reflective and can internalize professional and institutional values [7]. Measurements have been developed to identify the PIF stages of medical students, using questionnaires based on Kegan’s model [13, 14].

Numerous methods can assess professional identity development, including students’ reflections [8, 15] and a validated questionnaire. The latter can be used over time, which is critical for understanding dynamic development (e.g., Tagawa M, 2019 [13], Tagawa M, 2020 [14]). Studies on PIF among medical students are usually conducted by exploring this phenomenon in a given context, using focus group discussions on related topics [16]. Another way to study PIF is to measure students’ development through a professional identity essay (PIE) filled with responses to several prompts [17].

These approaches encourage students to consciously consider their professional identity development [15, 18]. For instance, personal narrative reflection encourages students to sense their current being as a student. This helps them reflect on any experience of identity dissonance and to narrate their future aspirations as healthcare professionals [7, 19]. Additionally, revealing the development of professional identity from the subconscious level generates further discussions with peers, senior colleagues, and medical teachers as mentors [7, 13]. Eventually, it can help students reshape and negotiate their professional identity.

Studies show that the professional development of medical students and residents, including misconduct in medical education, may predict future unprofessional behavior in practice [20, 21]. Further studies also highlighted unprofessional behaviors among medical students as caused by challenges in their reflectiveness and adaptability. Those behaviors require further identification of underlying problems, remediation and even case dismissal if necessary [22]. Furthermore, a study in a hierarchical and collectivist culture underscores the strong influence of the culture towards clinical year students’ responses when they encounter professional dilemma [23], showing that professionalism and professional identity formation are always contextual and should consider sociocultural backgrounds [24].

Therefore, as studies about professional identity formation of medical students have been extensively conducted in western contexts [e.g. 1–10], we would like to explore the PIF of medical students in Indonesia—a country with hierarchical and collectivist cultural backgrounds, in which the society accept inequality in power (superiority and subordinary) and prioritize on connectedness [25, 26]. Studies on PIF of medical teachers in this setting suggests the strong incorporation of religious values, family influences and societal recognition in their PIF [27]. PIF studies on medical students in this cultural setting, on the other hand, is rather limited.

Our study therefore is expected to yield further information in this specific context regarding the roles of individuals and institutions in PIF of medical students. In addition, studies aiming to measure PIF using a quantitative tool and to explore it using qualitative approaches are usually conducted separately. Therefore, considering the role of medical schools and the importance of PIF for medical students, we would like to measure PIF at various stages of medical training and explore students’ perceptions of PIF and its contributing/inhibiting factors sequentially. Our research questions are three-folds: a. What is the validity of an adapted PIF questionnaire in Indonesia context?; b. What are the measures of PIF of students at various stages of medical education?; c. How do students perceive PIF and its contributing/inhibiting factors? The validation and use of instruments to measure PIF can further support professional development in medical students through the identification of ‘where they are at’ and the exploration of contributing/inhibiting factors are expected to further inform medical schools to support and nurture the PIF contextually.

## Method

### Context

The study was conducted at Faculty of Medicine Universitas Indonesia, a medical school that is home to more than 40 undergraduate and postgraduate medical programs. The undergraduate program has the largest numbers of students among other programs. Students of the undergraduate and postgraduate programs involved in this study were enrolled in a competency-based medical curriculum in their respective programs.

### Design

This is a mixed-methods study using a sequential explanatory approach [28]. We selected this approach to gain a comprehensive understanding of PIF among medical students and residents, using systematic quantitative and qualitative measures, as PIF is a complex phenomenon that requires a deep and reflective understanding of its stage and dynamic processes [17]. The purpose of using mixed methods approach in this study was to provide a more thorough qualitative description to explain further about the findings from the quantitative stage [29]. While the quantitative measurement of PIF using validated questionnaire in this study was aimed to provide the PIF profiles of medical students across study years, further exploration through FGDs was intended to provide the dynamic processes of the PIF.

### Respondents

The study involved undergraduate medical students (years 2, 4, and 6) and postgraduate medical students or residents (years 2–3 of each program). Their involvement at these levels was expected to facilitate the study aim of observing differences in PIF questionnaire scores to better understand PIF among students at different year levels.

### Quantitative stage

a. Instrument.

The questionnaire in this study was adapted from a questionnaire developed by Tagawa [13, 14]. The use of the questionnaire was supported by the construct validity and good reliability of the questionnaire which is aligned with the PIF stages of medical students based on Kegan’s model. The opportunity to capture the different levels of PIF using the questionnaire was critical in this study. This was translated from English into Indonesian and back-translated to assure meaning comparability and content validity (see Table 1). The translations were completed by a professional translator and curated by the authors (AF, EF, NG), who have expertise in medical education and have been studying PIF in medical education. Following translation, we used the questionnaire to complete a cognitive interview involving three authors (TZA, MAF, and MF), who are current undergraduate medical students at FMUI, as well as 5 other students who did not participate in the survey. Subsequent amendments to the Indonesian translation were made to relevant items to improve clarity and facilitate appropriate responses. Each revision involved a back-translation amendment and check of the meaning comparability with the original items.

### b. Data collection

In the first stage of the study, we administered the translated PIF questionnaires developed by Tagawa [13, 14] to undergraduate medical students year two, four, and six and residents year two. We involved residents year two in this study as they resembled groups of practicing doctors so that we could explore the PIF process more comprehensively. We employed a total sampling approach, with a target response rate of 60–70% of each group of respondents. All potential respondents were invited through an online broadcast via the group leaders and study program coordinators. It was emphasized to the potential respondents that their participation was voluntary and would not affect their ongoing study and evaluation. Data collection was completed from August–September 2020, and several reminders were sent out to increase the response rate.

After administering the questionnaire, using SPSS IBM 27 we completed exploratory factor analysis (EFA) to identify latent variables in the questionnaire by developing factors or dimensions constructed by strongly correlated items [30]. We aimed to compare the factors identified in our analysis to those in the original questionnaire [13, 14]. We conducted an EFA with Principal Axis Factoring (PAF) [31, 32] to support the construct validity of our PIF questionnaire.

Following the instrument validation, further data analysis was conducted to compare and contrast the scores of PIF among the four groups of respondents. Given the abnormal distribution of the data, non-parametric tests, Kurskall-Wallis were completed to analyze the median difference of the four groups, followed by Mann–Whitney as a post-hoc analysis).

### Qualitative stage

Following the data analysis of the quantitative phase*,* focus group discussions were conducted to further explore the findings, particularly to explore the perceptions in regards to the PIF process and the factors that contribute and inhibit it. Focus group questions were developed based on a concept of PIF integrated with socialization theory, as well as Kegan’s model [7] (Appendix 2).

In order to best represent views on PIF, focus group (FG) participants were purposively selected using maximum variety sampling approach from those who filled out the questionnaires and agreed to be invited to a focus group session [29]. The maximum variety sampling approach was used to select the FG participants, accounting for representativeness regarding gender and study program (for residents). Two FGs were conducted for each class group (years 2, 4, and 6) of undergraduate program; two other FGs were conducted for residents in both surgical and nonsurgical study programs.

All FGs were moderated by the core research teams who were medical educationalists in the institution with no involvement in the assessment process of students or residents participating in the FGs. All FGs were conducted online using video conference platforms due to the COVID-19 pandemic ongoing at time. Focus groups were recorded in the platform for further analysis.

The qualitative data obtained were transcribed verbatim and analyzed using a thematic analysis using inductive and deductive approach according to the related theory of PIF using the Steps for Coding and Theorisation (SCAT) method [33]. The initial thematic analysis followed by initial discussion to identify the core themes and subthemes was conducted on two transcripts independently by two authors who were also the FG moderators [AF and NG] prior to further analysis of all transcripts. The study was approved by the Research Ethical Committee of the Faculty of Medicine Universitas Indonesia (Number: KET-497/UN2.F1/ETIK/PPM.00.02/2020).

## Results

### Questionnaire validation

We performed EFA using PAF with oblique rotation. The EFA contains several steps. First, our analysis using the Kaiser–Meyer–Olkin (KMO) and Bartlett’s Test of Sphericity showed that the data fulfilled the initial criteria of the EFA (KMO = 0.831 and Bartlett’s Test of Sphericity = X2 1.357 (105), p 0.000). Second, the questionnaire items were correlated, factors were extracted, and oblique rotation was conducted. The eigenvalue and scree plot were used to determine the number of retained factors (Appendix 1). All items were loaded strongly (> 0,4), especially in one factor. There were no cross-loadings and each factor consisted of at least 3 items, leading to adequate support for the constructed factors [31, 32] (Table 1). The interpretation of conceptual meaning of the constructed factors resulted in 4 factors/subscales: recognition and internalization of professional roles (items 8, 9, 12, 14); self-control in professional behavior (items 3, 6, 7, 15); reflections on professionalism (items 10, 11, 13); and thought processes as a medical/health professional (items 1, 2, 4, 5).

The Cronbach’s alphas of the overall scale and each subscale were calculated to assess the internal consistency of the questionnaire. The reliability of the overall scale was 0.776, while that of subscales 1–4 were 0.662, 0. 661, 0.627, and 0.522, respectively; these are quite satisfactory results, with the exception of subscale 4 [34].

**Table 1:** Exploratory Factor Analysis – Rotated Matrix

No	Items	Component			
		1	2	3	4
1	I cannot tolerate that colleagues who sympathize with my actions have a different mindset from me *Saya tidak bisa mentoleransi kolega yang memiliki pola pikir yang berbeda dari saya namun bersimpati terhadap tindakan saya*	.107	-.019	-.164	**.707**
2	I find it difficult to suppress my desires and act rationally *Sulit bagi saya untuk menyembunyikan keinginan dan bertindak secara rasional*	.036	.086	-.132	**.678**
3	It is difficult for me to adjust and act according to the different values of each medical professional and the demands for physicians *Sangat sulit bagi saya untuk menyesuaikan dan bertindak sesuai dengan nilai dan tuntutan profesi dokter*	.125	**.628**	.103	.375
4	I have never thought about the reasons or principles behind the required code of conduct *Saya tidak pernah berpikir mengenai alasan atau prinsip di balik kode etik yang perlu dilaksanakan*	.004	.257	.208	**.470**
5	I am sometimes unable to do something I was not interested in despite understanding its necessity *Saya terkadang enggan melakukan sesuatu yang tidak saya minati, walaupun saya mengerti pentingnya hal tersebut*	-.020	.373	.289	**.470**
6	The way I behave in medical settings is not my true self *Cara saya bertindak di dunia medis bukan merupakan representasi diri saya sebenarnya*	.022	**.598**	.229	.280
7	I behave correctly as a physician on a daily basis *Dalam kehidupan sehari- hari, saya berperilaku sebagai seorang dokter dengan benar*	.579	**.588**	.143	-.019
8	I am aware of my position as a physician *Saya sadar posisi saya sebagai seorang dokter*	**.751**	.366	.051	.016
9	I have accepted the words of gratitude and the frustration and anger of patients as a personal evaluation of myself *Saya menerima ucapan terima kasih, rasa frustrasi dan amarah pasien sebagai bahan evaluasi pribadi diri saya*	**.752**	-.060	.174	.075
10	I consider long-term significance and concerns when I think about what I should do now *Saya mempertimbangkan kepentingan dan perhatian jangka panjang saya saat memikirkan apa yang harus saya lakukan sekarang*	.221	.340	**.594**	.024
11	I have used my own beliefs and ideals as a standard to evaluate my own actions as a physician *Saya menggunakan kepercayaan dan idealisme saya sebagai standar untuk mengevaluasi perilaku saya sebagai seorang dokter*	.239	.083	**.722**	-.067
12	If I were able to play a role in improving society and organizations, I would be satisfied even if I did not receive individual recognition *Apabila saya dapat berperan dalam memperbaiki masyarakat dan organisasi, saya akan puas walaupun tidak mendapatkan pengakuan individu*	**.458**	-.203	.277	.360
13	I induce action in the people around me based on the principles I believe in to fulfill my role as a physician *Saya mendorong orang-orang di sekitar saya untuk bertindak berdasarkan prinsip yang saya yakini untuk memenuhi peran saya sebagai seorang dokter*	.173	-.098	**.744**	-.015
14	I take on various roles in accordance with the demands of society *Saya mengambil berbagai peran yang sesuai dengan kebutuhan masyarakat*	**.627**	.108	.317	.035
15	I feel that I need to change my current mindset and everyday behavior *Saya merasa bahwa saya harus mengubah pola pikir dan perilaku sehari-hari saya*	.063	**.709**	-.137	-.049

### Quantitative stage

A total of 443 respondents participated in the survey stage, with 106 (23.9%), 110 (24.8%), 108 (24.4%), and 119 (26.9%) participants in year 2, year 4, year 6, or stage 2 (residents), respectively. The response rate at each level was 46–71% of total respondents in each group. Univariate analysis of the questionnaire is described in Table 2.

**Table 2:** The questionnaire’s response distribution

No	Item description	Item score (mean (SD))			
		Undergraduate			Postgraduate-residents
		Preclinical year 2	Preclinical year 4	Clinical year 6	
1	I cannot tolerate that colleagues who sympathize with my actions have a different mindset from me *Saya tidak bisa mentoleransi kolega yang memiliki pola pikir yang berbeda dari saya namun bersimpati terhadap tindakan saya*	5.44 (1.160)	5.35 (1.309)	5.27 (1.280)	5.34 (1.217)
2	I find it difficult to suppress my desires and act rationally *Sulit bagi saya untuk menyembunyikan keinginan dan bertindak secara rasional*	4.79 (1.485)	5.25 (1.468)	5.06 (1.439)	5.07 (1.522)
3	It is difficult for me to adjust and act according to the different values of each medical professional and the demands for physicians *Sangat sulit bagi saya untuk menyesuaikan dan bertindak sesuai dengan nilai dan tuntutan profesi dokter*	4.96 (1.279)	5.53 (1.155)	5.77 (1.056)	6.16 (0.833)
4	I have never thought about the reasons or principles behind the required code of conduct *Saya tidak pernah berpikir mengenai alasan atau prinsip di balik kode etik yang perlu dilaksanakan*	5.28 (1.392)	5.41 (1.258)	5.50 (1.264)	5.50 (1.333)
5	I am sometimes unable to do something I was not interested in despite understanding its necessity *Saya terkadang enggan melakukan sesuatu yang tidak saya minati, walaupun saya mengerti pentingnya hal tersebut*	3.95 (1.558)	3.74 (1.519)	3.98 (1.559)	4.51 (1.425)
6	The way I behave in medical settings is not my true self *Cara saya bertindak di dunia medis bukan merupakan representasi diri saya sebenarnya*	4.86 (1.355)	5.00 (1.440)	5.19 (1.517)	5.76 (1.340)
7	I behave correctly as a physician on a daily basis *Dalam kehidupan sehari- hari, saya berperilaku sebagai seorang dokter dengan benar*	4.34 (1.086)	4.47 (1.081)	5.06 (1.022)	5.87 (0.965)
8	I am aware of my position as a physician *Saya sadar posisi saya sebagai seorang dokter*	5.07 (1.165)	5.18 (1.265)	5.79 (0.786)	6.33 (0.702)
9	I have accepted the words of gratitude and the frustration and anger of patients as a personal evaluation of myself *Saya menerima ucapan terima kasih, rasa frustrasi dan amarah pasien sebagai bahan evaluasi pribadi diri saya*	5.21 (1.110)	5.33 (1.085)	5.81 (0.88)	5.78 (0.976)
10	I consider long-term significance and concerns when I think about what I should do now *Saya mempertimbangkan kepentingan dan perhatian jangka panjang saya saat memikirkan apa yang harus saya lakukan sekarang*	5.46 (1.367)	5.57 (1.079)	5.66 (1.078)	5.92 ( 0.783)
11	I have used my own beliefs and ideals as a standard to evaluate my own actions as a physician *Saya menggunakan kepercayaan dan idealisme saya sebagai standar untuk mengevaluasi perilaku saya sebagai seorang dokter*	5.12 (1.048)	5.33 (1.126)	5.40 (1.076)	5.58 (1.204)
12	If I were able to play a role in improving society and organizations, I would be satisfied even if I did not receive individual recognition *Apabila saya dapat berperan dalam memperbaiki masyarakat dan organisasi, saya akan puas walaupun tidak mendapatkan pengakuan individu*	5.52 (1.189)	5.21 (1.369)	5.24 (1.282)	5.52 (1.119)
13	I induce action in the people around me based on the principles I believe in to fulfill my role as a physician *Saya mendorong orang-orang di sekitar saya untuk bertindak berdasarkan prinsip yang saya yakini untuk memenuhi peran saya sebagai seorang dokter*	4.58 (1.294)	4.63 (1.248)	4.85 (1.198)	4.65 (1.453)
14	I take on various roles in accordance with the demands of society *Saya mengambil berbagai peran yang sesuai dengan kebutuhan masyarakat*	4.76 (1.109)	4.8 (1.233)	4.92 (1.015)	5.24 (1.102)
15	I feel that I need to change my current mindset and everyday behavior *Saya merasa bahwa saya harus mengubah pola pikir dan perilaku sehari-hari saya*	2.95 (1.341)	3.21 (1.447)	3.62 (1.445)	4.38 (1.378)

Table 3 presents further analyses of the score comparisons for undergraduate medical students in years 2, 4, and 6, as well as stage 2 residents.

**Table 3:** PIF questionnaire scores (*N* = 443)

	**N**	**Mean**	**SD**	**Median**	**Range**	
**PIF total score**						
Undergraduate—Preclinical year 2	106	63,81	7,55	63,00	47—86	Kruskal Wallis test *p* = 0,272
Undergraduate—Preclinical year 4	110	63,04	6,61	63,00	45–85	
Undergraduate—Clinical year 6	107	64,35	5,67	65,00	46–85	
Postgraduate—Residency program	120	64,15	6,42	64,00	48–83	
**PIF subscale 1 score**						
Undergraduate—Preclinical year 2	106	20,56	3,33	20,00	12–28	**Kruskal Wallis test *p* = 0,000**
Undergraduate—Preclinical year 4	110	20,52	3,23	21,00	12–28	
Undergraduate—Clinical year 6	107	21,79	2,69	22,00	13–28	
Postgraduate—Residency program	120	22,83	2,98	23,00	13–28	
**PIF subscale 2 score**						
Undergraduate—Preclinical year 2	106	17,11	3,36	17,00	8–27	**Kruskal Wallis test** ***p*** **= 0,000**
Undergraduate—Preclinical year 4	110	18,21	3,16	18,00	10–24	
Undergraduate—Clinical year 6	107	19,67	3,45	20,00	11–27	
Postgraduate—Residency program	120	22,1	3,08	22,00	12–28	
**PIF subscale 3 score**						
Undergraduate—Preclinical year 2	106	15,16	2,82	15,00	8–21	Kruskal Wallis test *p* = 0,107
Undergraduate—Preclinical year 4	110	15,53	2,67	15,00	9–21	
Undergraduate—Clinical year 6	107	15,92	2,52	16,00	6–21	
Postgraduate—Residency program	120	16,14	2,64	16,00	9–21	
**PIF subscale 4 score**						
Undergraduate—Preclinical year 2	106	19,47	3,87	20,00	9–27	Kruskal Wallis test *p *= 0,290
Undergraduate—Preclinical year 4	110	19,74	3,51	20,00	10–28	
Undergraduate—Clinical year 6	107	19,81	3,43	20,00	12–28	
Postgraduate—Residency program	120	20,42	3,51	21,00	11–28	

Table 3 shows a total PIF score difference among the 4 groups and indicates that year 6 students and stage 2 residents had higher scores than the other two groups, although these differences are not statistically significant. The significant subscale score differences are observed in subscales 1 and 2 regarding “Recognition and internalization of professional roles” and “Self-control towards professional behaviors”, respectively. The post-hoc analysis using the Mann–Whitney test results is described as follows, with the use of adjusted Bonferroni p [35]:

- a. Post-hoc analysis of subscale 1.
  Year 2 and stage 2 residents; X2 12.689, adjusted Bonferroni p 0.002
  Year 4 and year 6 students; X2 9.296, adjusted Bonferroni p 0.014
  Year 4 students and stage 2 residents; X2 30.689, adjusted Bonferroni p 0.000
  Year 6 students and stage 2 residents; X2 7.630, adjusted Bonferroni p 0.034
- b. Post-hoc analysis of subscale 2.
  Year 2 and year 6 students; X2 23.696, adjusted Bonferroni p 0.000
  Year 2 students and stage 2 residents; X2 71.768, adjusted Bonferroni p 0.000
  Year 4 and year 6 students; X2 7.010, adjusted Bonferroni p 0.049
  Year 4 students and stage 2 residents; X2 60.964, adjusted Bonferroni p 0.000
  Year 6 students and stage 2 residents; X2 15.753, adjusted Bonferroni p 0.000

### Qualitative Stage

We conducted eight focus group discussions involving a total of 69 participants. Table 4 shows the number of participants in each focus group.

**Table 4:** Focus groups participants

Year groups	Focus Groups	Number of participants	Male participants	Female participants
Year 2	FGD 1	10	5	5
	FGD 2	8	3	5
Year 4	FGD 1	12	7	5
	FGD 2	9	5	4
Year 6	FGD 1	9	7	2
	FGD 2	9	6	2
Residents	Non-surgical	8	4	4
	Surgical	4	-	4

**Fig. 1:**
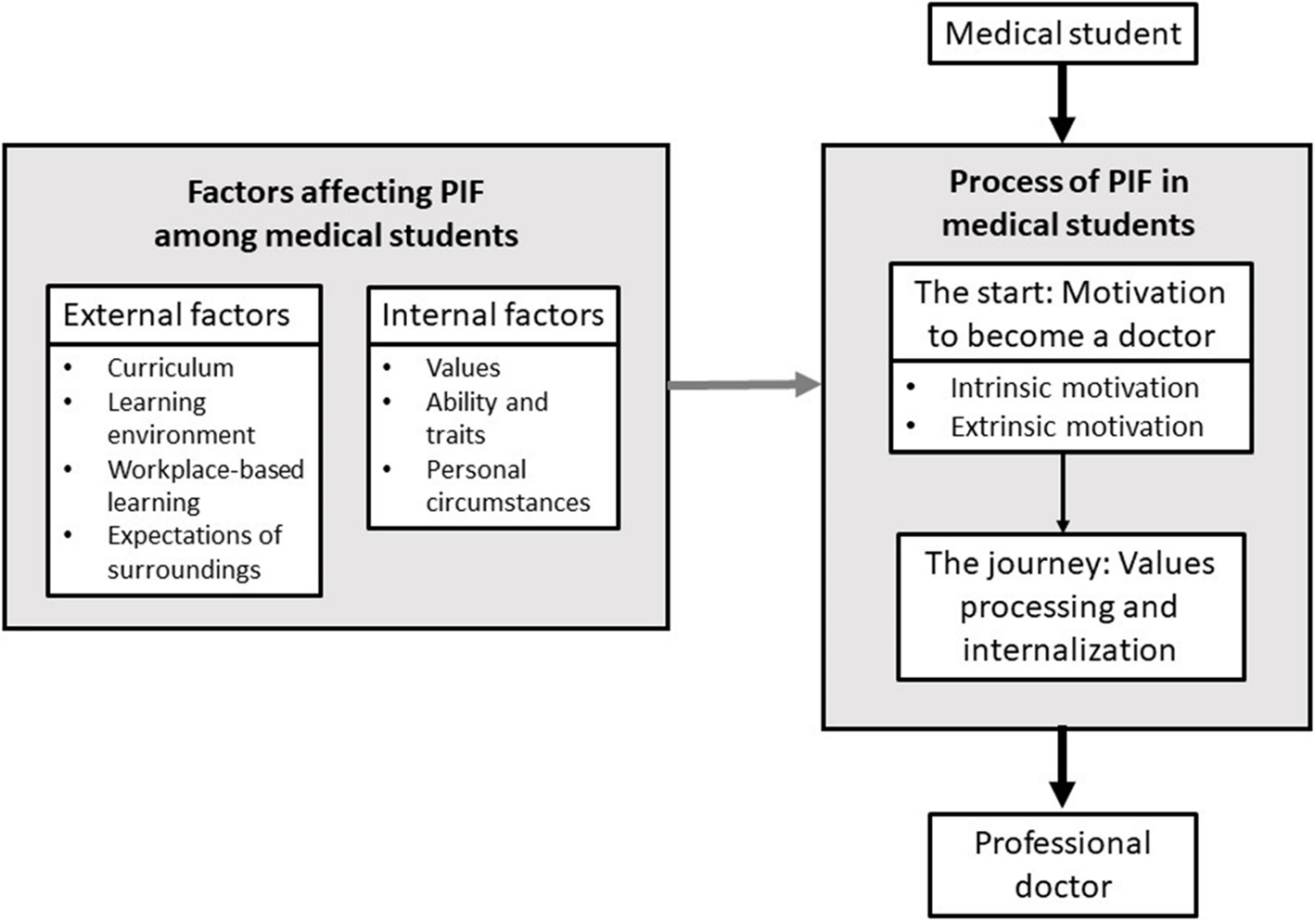
Relationships of the themes and subthemes

Two themes emerge from the focus groups, depicting the process of PIF in medical students and the factors that affect it. The relationships of the themes and subthemes are described in Fig. 1.

1. **Process of PIF in medical students**

#### How it started: motivation to become a doctor

### Intrinsic Motivation

The students reported both intrinsic and extrinsic motivations for becoming doctors. Their reasons for choosing medicine varied. The most common motivation was a desire to help others:

*“I have always felt that the purpose of my existence is to help others. And the way to do that is by becoming a doctor. At first, I wanted to be an engineer, but later I realized that the impact that doctors can have on other people’s lives is more profound.” -R, second-year student.*

This realization about doctors helping people stems from personal experience in the family or close social circles:

*“Since I was in secondary school, I have decided that I want a profession that will not just provide for me financially, but that will also let me help other people, and I will get the reward later in the afterlife.” -D, non-surgical resident.*

Some students mentioned their interest in medicine-related subjects (mostly biology) during their school years. They were curious about these subjects and wished to learn more in college:

*“I really like biology. I used to look for medicine-related information, which made me even more curious. I want to know how the human body works.” -P, second-year student*

### Extrinsic Motivation

The presence of role models in their lives seemed to influence students to choose medicine as their path. These role models were typically family members or a doctor with whom they interacted when they or their family required medical care.

*“My parents are doctors, so I was introduced to their field of work when I was little. I really look up to my dad and I want to be like him. When he came home from work at night, he used to share his stories about the cases he had, and I found them very interesting.” -R, second-year student.*

Being a doctor comes with advantages, such as being seen as knowledgeable and having an important position in the community; these also play a role in motivating students to pursue this career.

*“I come from a small town, and we only have one pediatrician. I can see how this doctor is highly respected and well-known by the people.. it really looked like he is the community leader there, and I think I want to be a doctor because of that.” -I, final-year student.*

Many parents want their children to be doctors, which constitutes another important motive:

*“I was not really interested in being a doctor at first, but my mom really wants her son to be a doctor. So my motivation is to make my mom happy.” -A, second-year student*

#### The journey: how the values were processed and internalized

PIF consists of formation and internalization processes. Students reported experiencing PIF throughout their education. This process occurred gradually as they progressed, as reflected in their answers when they were asked to compare their current selves to the ideal picture of the doctors they wanted to become:

*“When I first read about the competencies and 7-star doctors, I knew that it was a very high standard to achieve. Now I feel that I am still very far from that. But I believe that we are all a ‘work in progress’; we need to practice lifelong learning. Admitting that we are still lacking is necessary to drive us to learn more. This is what makes medicine a unique and ever-evolving profession.” -E, sixth-year student.*

PIF is a continuous and dynamic process, with various factors supporting and inhibiting it. As students became further involved, they grew to recognize the complexity of the process.

*“I realize that the process that I am going through now is part of the learning. We can never tell what we might become unless we put forth our best effort and just finish the task. Even though it can be hard at times, just try to do it anyway.” -R, fourth-year student.*

During the PIF process, students’ wellbeing is another important factor. In fact, students perceived wellbeing as an essential quality of the ideal doctor:

*“A good doctor should be able to manage their life and find balance between their profession and their family life.” -D, non-surgical resident*

*“I have come to realize the importance of taking care of myself. I started reflecting on how I could encourage people to live a healthy life if I myself did not practice it.” -D, fourth-year student*

2. **Factors affecting PIF among medical students**

#### Internal factors

We identify three internal factors that may affect PIF among medical students: values, abilities and traits, and personal circumstances.

### Values

Values constitute an internal factor that promotes PIF in medical students. For example, integrity and responsibility were mentioned as supporting PIF:

*“In my opinion, [to be a good doctor], it is important to have these three things: 1. Loyalty—being committed to what we are doing; 2. responsibility for what we say and do; and 3. dependability.” -H, second-year student.*

Additionally, the internal values of 7-star doctors—such as being an empathetic care provider and lifelong learner who continuously practices and models a healthy lifestyle—encourage the PIF process.

### Ability and Traits

Students mentioned several abilities (general or specific to clinical skills) as important for professional development. Clinical skills and those related to medical competencies were mentioned more frequently by clinical students and residents, while generic abilities were mentioned by participants across year groups:

*“Ideally, as medical doctors, we have to keep learning and not be easily satisfied with our current performance. And we should never look down on other doctors.” -Sk, resident*

These general abilities include the ability to collaborate, cope with challenges, regulate emotions, think critically, ask questions, adapt, and maintain a work–life balance. Participants also noted the importance of ability to regulate learning and seeking feedback towards professional development:

*“The need to ask questions in class is important because we might later see patients who ask similar questions; [as professionals], we should understand how [to explain] the progress of the disease.” -At, second-year student.*

### Personal Circumstances

Personal circumstances, such as whether a student is facing burnout or emotional exhaustion, affect the professional development process. Other personal qualities, such as self-expectations and a sense of competence, are also critical in professional development:

*“Many residents face burnout because of the workload and assignments. At those times, we just want to do something for ourselves and care less about others.” -R, resident*

*“I feel blessed and proud that—finally—what I’ve learned for years can be applied to patients, although only for simple cases. But it was enough, at least to know that I can handle [the patient’s problems] and know that what I’ve learned this entire time was not useless.” -S, final-year student.*

#### External factors

Some of the external factors affecting PIF are the curriculum, education system, learning environment, workplace-based learning, and external expectations.

#### Curriculum

Participants revealed that curriculum plays an important role in PIF. Burdensome tasks and assignments, one-way lectures, and monotonous teaching and learning methods can hinder professional development and dampen the desire for lifelong learning, particularly due to the protracted study period in medical education. This occurs more frequently during the transition stage, at the end of the preclinical stage. The score-oriented paradigm was also mentioned as an inhibiting factor in professional development, because it could damage students’ integrity:

“*I used to learn very diligently when I was in the first year. As time goes by, the learning materials are added up but the [teaching/learning] approaches are very similar. It feels like a repeating pattern, and what I usually do now is study only to pass the exam and avoid remedials.” -C, fourth-year student.*

Medical students also highlighted the importance of explicit teaching/learning activities and called for professionalism assessments to be integrated into the curriculum. Students also demanded a code of conduct regarding standardized professional and unprofessional behaviors and consistent practices among all stakeholders, as students felt that there was a discrepancy between what is taught and what is observed in daily practice.

“*As I progress through medical school, I see more differences between the rules being taught and the behavior of people, showing decreased levels of integrity.” -Q, final-year student.*

Because clinical skills are considered important, students value a curriculum that provides early clinical exposure during the preclinical stages. However, clinical students reported that clinical rotations conducted departmentally somewhat inhibited their professional development:

“*What I think makes it difficult to grasp the idea of being a professional medical doctor is that, in some clinical rotations, we only learn specifically about a particular discipline and pay less attention to the basic clinical skills and the clinical experiences [i.e., other patient aspects that are unrelated to the particular discipline].” -H, final-year student.*

Students reported that curriculum adaptations due to the COVID-19 pandemic (ongoing at the time of this study) created anxiety in their efforts to reach competency.

#### Learning Environment

In terms of the learning environment, students experienced situations in which things were not done exactly as they had been taught (hidden curriculum). In practice, numerous factors may preclude taking an ideal approach:

*“We realize that learning is not entirely about knowing the subject matter. As you become involved in patient care, you see that you need more than that. And sometimes what we are taught does not align with the reality in practice.” -E, final-year student.*

To deal with the complexity in the learning environment, medical students and residents emphasized the roles of teachers in their professional development. They value teachers as more authoritative figures who serve as good role models, provide feedback, and nurture students:

“*Through bedside teaching while examining patients, we observe how attendings communicate and treat patients, and we can adopt it. Also, when attendings observe us [and provide feedback], it really helps us to learn how to be professional.” -Ft, resident.*

Respondents also highlighted the importance of support systems from study program administrators, peers, and family. They also credited interactions through extracurricular activities and student organizations:

“*The student body and organizations are really important because they help us get used to managing time and interacting with many different kinds of people. It also helps us make a strong commitment and be responsible and professional.”-Sh, second-year student.*

The hierarchical nature of medical education often results in negative role modeling practices and bullying. The respondents described this issue as inhibiting their professional development process:

“*Interaction between senior and junior residents is not always smooth. Now that I am in [my seniors’ position], I finally understand the reason why they did what they did. But for me, I choose to do it differently.” -Yi, resident.*

#### Workplace-Based Learning

Preclinical students stated that they learned a lot about being professional during their shadowing sessions with teachers. Shadowing sessions were conducted in a module in which students were given an opportunity to shadow their clinical teachers in medical practice, thus visualizing their future occupation as medical doctors. Interacting with standardized patients also helped them develop their professional identity:

*“For me personally, the shadowing session really created a perception on what ideal and professional doctors were, and how they act.” -K, fourth-year student*

Clinical students mentioned the importance of interacting with patients during their clinical clerkship. The experience of learning in various hospitals and healthcare facilities helped them grasp not only their roles as medical doctors, but also the challenges commonly faced in the workplace:

“*Working as junior doctors in the primary health setting was really different from the theory being taught during these medical school years. And it gives me a clearer view that we are exposed to this situation so that we can always be professional regardless of the situation and limitations in the field.” -Y, final-year student.*

For residents, prior experience in working as general practitioners also helped them develop their professional identity:

*“It took me several years before finally deciding to continue with residency; I was an intern in a suburb area for one year, followed by working in a military hospital for another year, and then I moved to the city and worked in a private hospital for four years. Then I decided to continue with the residency program.” -F, resident.*

#### Expectations of Surroundings

In the process of internalizing professionalism, students paid attention to the expected behaviors of medical students from the community, as well as from their teachers:

“*I have to admit that one of the ways we learn is to follow our teachers’ expectations. Sometimes it was good because it resembles the ideal doctor, but sometimes we only try to please the teacher, not to understand the knowledge itself.” -H, final-year student.*

## Discussion

Our research on PIF among medical students utilizes a mixed-methods (sequential explanatory) approach to measure the development of PIF at various stages of medical education. We explore students’ perceptions of PIF, along with its contributing and inhibiting factors. To measure PIF, we have adapted a validated questionnaire by Tagawa (2019, 2020) and further analyzed it systematically to provide evidence for its content and construct validity and reliability. Thus, our use of the questionnaire in this setting was justified and supported by robust instrument preparation [13, 14]. The four factors identified from the EFA—recognition and internalization of professional roles, self-control in professional behavior, reflections on professionalism, and thought processes as a medical/health professional—align with the PIF conceptual frameworks [7, 8].

Tagawa’s original questionnaire (Tagawa 2019, Tagawa 2020) consists of 15 items categorized into 5 factors: self-control as a professional, awareness of being a medical doctor, reflection as a medical doctor, execution of social responsibility, and external and internal self-harmonization [13, 14]. These factors align with our EFA results and with the PIF concept, despite some categorization differences. In our study, recognition and internalization of professional roles (Factor 1) encompasses items that carry direct meaning for how students internalize professionalism as part of becoming medical doctors. The second factor, self-control in professional behavior, includes examples of how medical students control themselves upon encountering potential emotional or non-supportive conditions influencing their professional behavior. This factor and its items are similar to those in Tagawa's questionnaire. The third factor we identify (reflections on professionalism) is similar to Tagawa’s third factor (reflection as a medical doctor). Both factors include items related to aspects that affect self-evaluation, such as long-term effects and personal values. Unlike Tagawa’s questionnaire, the third factor in our study also included item 13, which further highlights personal values in reflections on professionalism. Meanwhile, item 9, which is included in Tagawa’s third factor, was identified as part of the first factor in our study (recognition and internalization of professional roles). Items identified as part of “thought process as a medical/health professional” (Factor 4) in this study were included under the factor “self-control as a professional” in Tagawa’s study.

Further analysis of the quantitative data reveals differences in subscale 1 (recognition and internalization of professional roles) and subscale 2 (self-control in professional behavior) among undergraduate medical students in years 2, 4, 6 and stage 2 residents; more advanced groups exhibited higher scores. Since it is expected that more experience and a better socialization process in medical education and healthcare would yield a more developed professional identity, this study demonstrates that the use of the PIF questionnaire supports this construct and may enable institutions to initially assess medical students’ PIF stage, before exploring and nurturing it further through various strategies [7, 8]. According to socialization theory [6], lower PIF questionnaire scores may reflect legitimate peripheral positions of the students in the community of practice as medical doctors. Students still bring in their personal identity, motivation, and family/friends’ influences. The higher the score, the more students transition into the community of practice towards full participation which highlights their increased capacity to ‘think and act’ like professionals. Medical schools have critical roles in nurturing the PIF of medical students since the socialization process can be facilitated through meaningful learning experience both in preclinical and clinical years, the availability of role models and mentors, explicit curriculum and assessment for professional development, students’ self-reflection skills and positive learning environment [7].

Aligned with the above discussion, the current study provides further explanation from the qualitative data revealed in the current setting, which depicts two primary themes: the process of PIF in medical students and the factors affecting PIF. First, the PIF process in medical students is initiated by students’ motivation to become medical doctors. This study reveals that a student's motivation can originate intrinsically or extrinsically, which further influences how students see themselves and their surroundings and how the interactions of multiple factors contribute to their goals and performances. The three key components elaborated in the Self-Determination (SDT) Theory—autonomy, relatedness, and competence [36]—further explain how motivations are central to the PIF process. Stronger intrinsic motivations have positive impacts on PIF, such as improved empathy development [37, 38].

Furthermore, the students in this study were aware that PIF is a journey; they saw themselves as progressing towards what they envision as their future selves as professional medical doctors. Despite the array of motivations identified in this study, students from various year levels and residents articulated a common vision of the attributes of professional medical doctors. Their visions spanned the necessary knowledge and skills, the need to become lifelong learners, the centrality of personal wellbeing, and the importance of people skills such as communication, teamwork, empathy, and self-awareness. This envisioning of what they are becoming, supported by intrinsic motivations, indicates the progression of their PIF [8].

In addition, we reveal that external factors play critical roles in the PIF process; these factors intercalate with internal factors in medical students, resulting in their professional development, showing a psychosocial transition [39]. Because students demonstrated awareness that PIF is a deliberate process and that they were currently in the transition stage, students should be supported so they can adapt successfully; these efforts should account for students’ personal circumstances, support systems, and learning strategies [39]. This is particularly emphasized in the current study which highlights the hierarchical and collectivist culture [25].

In this study, in line with the results of scoping review on undergraduate medical students’ PIF [12], students mentioned the curriculum (including the hidden curriculum), the learning environment, workplace-based learning, and expected behaviors from the surroundings as external factors affecting their PIF processes. These external factors affect the context in which the transition occurs [39]. Opportunities to have role models, interact with patients, and receive feedback were described as supportive in the PIF process. However, the practice of a hidden curriculum, as well as the ongoing pandemic, create uncertainty in this process. As the heart of PIF was to accept and adapt with changes, reflective interactions discussing this uncertainty have become crucial for the PIF process [40, 41]. Encouragement of such reflective inquiries by students is necessary as it would facilitate their learning and their ability to take advantage for their professional development, even from the negative role modelling [42]. Coming from a culture where uncertainty tends to be highly avoided, this study underscores the importance of a more teacher-driven, structured, longitudinal approach, providing clear guidance and guidelines for students, to conduct reflective practice and in seeking feedback from their mentors [12, 23].

Ideally, these external factors can be addressed with support throughout the transition, for example, by ensuring the availability, quality, and relevance of an institutional support network for students [39]. The results of our study show that some of these external factors hinder more than support the process. For example, the informants in this study suggest that the interaction between senior-junior students and between teacher and students can be very hierarchical and not always constructive towards their understanding of their roles and professional development. Considering that identity is dynamic and that PIF among medical students is highly influenced by informal out-of-classroom interactions with other members of the professions, educational institutions should provide adequate network and institutional support for PIF through more casual interactions in various learning settings, in order to foster successful transformations [43, 44].

This study has several implications for medical education and the professional development of medical students and residents, in particular in the collectivist and hierarchical setting. First, PIF is an active and longitudinal process that requires motivation to control the dialogue of internal and external factors within oneself. Therefore, student-centered and personalized learning opportunities enriched with reflection and mentoring are necessary in medical schools to nurture motivation and a positive PIF process. Our study shows the importance of motivation and other personal factors, such as coping mechanisms, in students’ resilience in their medical education endeavor, which is instrumental for PIF [45, 46]. Second, medical students must consider what they are becoming and where they are in this process. Consequently, measurements of PIF using a questionnaire, as applied in this study, can be a useful metric for assessing PIF among medical students. Of course, this approach should be implemented alongside further reflective discussions to help students gain a meaningful understanding of their PIF process. Third, given the role of external factors, we also highlight the need to modify and optimize curricula to support PIF where appropriate, such as by incorporating more interactive teaching/learning sessions, integrated and relevant clinical rotations, positive role-modeling, early workplace-based learning, and feedback and mentoring in the preclinical and clinical stages.

We acknowledge the limitations of this study. It was conducted in a single institution, which may limit its generalizability, given some contextual factors. We involved medical students from different year levels and adopted a mixed-methods approach to elucidate the PIF profiles of medical students and perform in-depth analysis of its nature and contributing factors; we hope that our findings will prove relevant in other settings. In addition, the first stage of this study employed a cross-sectional approach. Therefore, although this study found some significant differences in PIF subscales (with higher scores for higher-level students), it could not elaborate the actual PIF development. Further studies should utilize the questionnaire longitudinally to assess PIF over time and document the narratives of PIF at the respective levels.

## Conclusion

This study demonstrates a cross-cultural validity of the Tagawa PIF scales with modified four subscales: recognition and internalization of professional roles, self-control in professional behavior, reflections on professionalism and thought processes as a medical/health professional. Assessing PIF among medical students at different stages in a hierarchical and collectivist culture using the adapted PIF scales, our study demonstrates the PIF transition across educational stages, affected by internal factors (students’ values, abilities and traits, and personal circumstances) and external factors (curriculum, learning environment, workplace-based learning, and surroundings’ expectations). Therefore, an integrative approach in the curriculum to support PIF across educational stages is important so that students can optimize their inner potentials as well as their external learning opportunities during the PIF process. This study also highlights the importance of a conscious PIF process for medical students at different stages and reveals the need for further action from medical schools to assure longitudinal support for PIF.

## Notes on contributors

Ardi Findyartini, MD, PhD, is an Associate Professor in medical education. She is currently the Coordinator of Community Service, Department of Medical Education, Faculty of Medicine Universitas Indonesia and the Chair of Medical Education Cluster, Indonesia Medical Education and Research Institute, Faculty of Medicine Universitas Indonesia.

Nadia Greviana, DDS, MMedEd, is a Lecturer in medical education. She is currently the academic staff at the Department of Medical Education, Faculty of Medicine Universitas Indonesia and the core researcher at the Medical Education Cluster, Indonesia Medical Education and Research Institute, Faculty of Medicine Universitas Indonesia.

Estivana Felaza, MD, MMedEd, is a Senior Lecturer in medical education. She is currently the academic staff at the Department of Medical Education, Faculty of Medicine Universitas Indonesia and the core researcher at the Medical Education Cluster, Indonesia Medical Education and Research Institute, Faculty of Medicine Universitas Indonesia.

Muhammad Faruqi, is currently a fourth-year medical student at the Undergraduate Medical Program Faculty of Medicine Universitas Indonesia and a student intern at the the Medical Education Cluster, Indonesia Medical Education and Research Institute, Faculty of Medicine Universitas Indonesia.

Taris Zahratul Afifah, is currently a third-year medical student at the Undergraduate Medical Program Faculty of Medicine Universitas Indonesia and a student intern at the the Medical Education Cluster, Indonesia Medical Education and Research Institute, Faculty of Medicine Universitas Indonesia.

Mutiara Auliya Firdausy, is currently a fourth-year medical student at the Undergraduate Medical Program Faculty of Medicine Universitas Indonesia and a student intern at the the Medical Education Cluster, Indonesia Medical Education and Research Institute, Faculty of Medicine Universitas Indonesia.

## Supplementary Information

**Additional file 1.**

## Data Availability

The datasets generated and/or analysed during the current study are not publicly available due to conditions of participants’ consents but are available from the corresponding author on reasonable request.
