# Supplementary material for: Professional identity formation of medical students: A mixed-methods study in a hierarchical and collectivist culture
Source: BMC Med Educ. 2022 Jun 8;22:443. doi: 10.1186/s12909-022-03393-9 (PMC9175156; doi:10.1186/s12909-022-03393-9)
Supplement: Supplementary file 1 — Additional file 1. [file 12909_2022_3393_MOESM1_ESM.docx]

**Appendix 1**

Table. Eigenvalue analysis of the extracted components/factors

| **Component** | **Initial Eigenvalues** | | | **Extraction Sums of Squared Loadings** | | | | | **Rotation Sums of Squared Loadings** | | |
| --- | --- | --- | --- | --- | --- | --- | --- | --- | --- | --- | --- |
|  | Total | % of Variance | Cumulative % | Total | | % of Variance | Cumulative % | Total | | % of Variance | Cumulative % |
| **1** | **3.866** | **25.776** | **25.776** | **3.866** | | **25.776** | **25.776** | **2.236** | | **14.907** | **14.907** |
| **2** | **1.877** | **12.510** | **38.286** | **1.877** | | **12.510** | **38.286** | **2.137** | | **14.244** | **29.151** |
| **3** | **1.252** | **8.346** | **46.633** | **1.252** | | **8.346** | **46.633** | **1.911** | | **12.739** | **41.890** |
| **4** | **1.054** | **7.026** | **53.658** | **1.054** | | **7.026** | **53.658** | **1.765** | | **11.768** | **53.658** |
| 5 | .894 | 5.963 | 59.621 |  | |  |  |  | |  |  |
| 6 | .781 | 5.205 | 64.827 |  | |  |  |  | |  |  |
| 7 | .752 | 5.016 | 69.843 |  | |  |  |  | |  |  |
| 8 | .710 | 4.734 | 74.577 |  | |  |  |  | |  |  |
| 9 | .659 | 4.395 | 78.972 |  | |  |  |  | |  |  |
| 10 | .645 | 4.302 | 83.273 |  | |  |  |  | |  |  |
| 11 | .605 | 4.035 | 87.308 |  | |  |  |  | |  |  |
| 12 | .564 | 3.759 | 91.067 |  | |  |  |  | |  |  |
| 13 | .514 | 3.425 | 94.492 |  | |  |  |  | |  |  |
| 14 | .473 | 3.151 | 97.643 |  | |  |  |  | |  |  |
| 15 | .354 | 2.357 | 100.000 |  | |  |  |  | |  |  |
| Extraction Method: Principal Component Analysis. | | | | |  |  |  |  | |  |  |


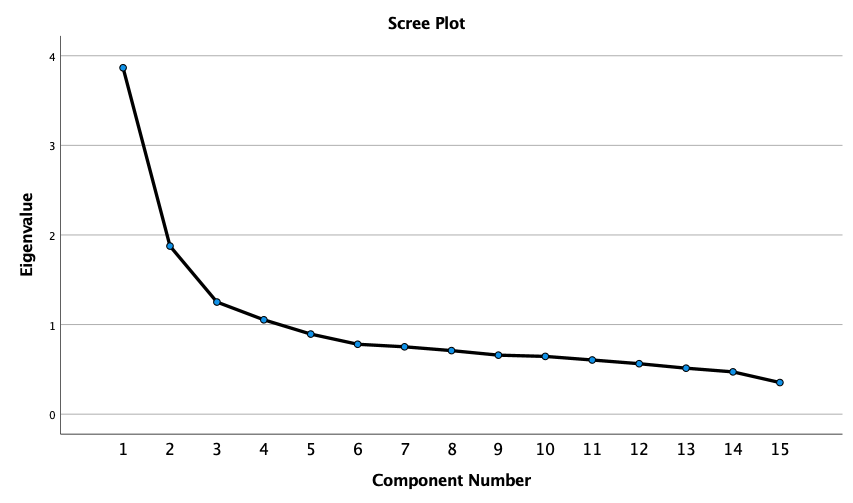


Figure. Screeplot showing support of 4 components/factors retain

Appendix 2

FGD questions

*Opening*

1. How do you currently feel as medical student/ resident in this stage?
2. How do you describe your current educational stage? Please explain

*Core questions*

1. What motivates you to become medical doctor?
2. How do you describe ideal doctors? (Probing: in knowledge, skills, behaviour)
3. How do you perceive yourself, do you feel ready to become a professional medical doctor? Why?
4. Can you describe a moment that affect your learning to become professional medical doctor?
5. How do you learn to be professional?
   1. What factors support you to be professional?
   2. What factors hinder you to be professional?
   3. Probe: curriculum, content, interaction, assessment, etc

*Closing*

1. If you became an authority in medical school or teaching hospital, what would you change so that it supported your process to become professional?
